# Dietary Fat Content Influences PanIN Progression and Pancreatic Cancer Development in Mice

**DOI:** 10.1158/2767-9764.CRC-25-0777

**Published:** 2026-05-05

**Authors:** Urvinder Kaur Sardarni, Erika Y. Faraoni, Alyssa M. Waller, Lincoln N. Strickland, Baylee O’Brien, Kelsey A. Klute, Jesse L. Cox, Florencia McAllister, Jennifer M. Bailey-Lundberg

**Affiliations:** 1 Department of Pathology, Microbiology and Immunology, University of Nebraska Medical Center, Omaha, Nebraska.; 2 Fred and Pamela Buffett Cancer Center, University of Nebraska Medical Center, Omaha, Nebraska.; 3 Department of Genetics, University of Texas MD Anderson Cancer Center, Houston, Texas.; 4 The Eppley Institute for Research in Cancer and Allied Diseases, University of Nebraska Medical Center, Omaha, Nebraska.; 5 Texas A&M Medical School, College Station, Texas.; 6 Department of Internal Medicine, University of Nebraska Medical Center, Omaha, Nebraska.; 7 GI Medical Oncology, The University of Texas MD Anderson Cancer Center, Houston, Texas.

## Abstract

Dietary macronutrient composition has emerged as a key modulator of pancreatic tumorigenesis, yet the impact of lipid-rich diets, particularly ketogenic diets (KD), on the earliest stages of pancreatic cancer development remains unclear. To investigate how dietary lipids shape the initiation and progression of *Kras*-driven neoplasia, we examined the effects of a low-fat diet (LFD), high-fat diet (HFD), and KD in the *Ptf1a*^*CreERT2*^*;Kras*^*G12V*^ (*Acinar*^*KrasG12V*^) mouse model. KD-fed mice showed the shortest survival (median 26 ± 7 days) compared with the standard diet (SD, 87 ± 29; *P* = 0.02) and LFD (57 ± 27; *P* = 0.02), whereas HFD-fed mice also exhibited reduced survival relative to SD (35 ± 25; *P* = 0.05). KD feeding was associated with severe glucose intolerance and elevated circulating β-hydroxybutyrate levels. Histologically, KD-fed *Acinar*^*KrasG12V*^ mice developed invasive, sarcomatoid-like pancreatic ductal adenocarcinoma (PDAC), whereas HFD-fed mice showed increased poorly differentiated PDAC; in both groups, these aggressive tumors were associated with extensive fibrosis and increased stromal CD39 expression relative to tumor compartments. Proteomic analysis demonstrated activation of the PI3K–Akt–mTOR and EGFR signaling pathways in tumors from KD- and HFD-fed *Acinar*^*KrasG12V*^ mice. Serum cytokine/chemokine profiling revealed a proinflammatory and proangiogenic milieu in KD-fed *Acinar*^*KrasG12V*^ mice. Collectively, these results suggest that dietary lipid enrichment prior to oncogenic *Kras* activation is associated with accelerated early pancreatic neoplasia and a microenvironment conducive to tumor progression. These findings underscore the need for careful consideration of KD use in individuals at elevated risk for pancreatic cancer.

**Significance:**

This article evaluates the impact of KDs and HFDs prior to oncogenic *Kras* activation in the *Acinar*^*KrasG12V*^ model. These findings reveal that lipid-rich diets accelerate PDAC progression and have important implications for dietary recommendations in individuals at elevated pancreatic cancer risk.

## Introduction

Pancreatic ductal adenocarcinoma (PDAC), which accounts for more than 90% of all pancreatic cancer cases, is one of the most aggressive and lethal cancers and is projected to become the second leading cause of cancer-related deaths by 2030 (1). Dietary macronutrient composition has emerged as a key modifier of pancreatic tumorigenesis. In *Kras*^*G12D*^ mice, high-fat diet (HFD) feeding accelerates pancreatic intraepithelial neoplasia (PanIN) formation, enhances inflammation and desmoplasia, and reduces survival (2, 3). High-carbohydrate diets (HCD) exert similar but less pronounced effects, whereas high-protein diets show negligible impact on pancreatic neoplasia compared with controls (4). A recent study using inducible acinar-specific *Kras*^*G12V*^*/Trp53*-loss mice further demonstrates that HFD promotes inflammatory tumorigenesis and macrophage-mediated cathelicidin antimicrobial peptide–P2X purinoceptor 7 signaling, driving tumor plasticity and metastasis (bioRxiv 2026.02.10.703309). Interestingly, ketogenic diets (KD), composed of 80% to 90% fat, have demonstrated antitumor effects across several tumor types in both clinical and preclinical studies (5). By elevating circulating ketone bodies, KDs reprogram cellular metabolism toward fatty acid and ketone utilization while limiting glucose availability. As cancer cells rely heavily on aerobic glycolysis to sustain growth, restricting carbohydrates has been proposed as a metabolic strategy to suppress tumor progression (6). However, the impact of KD in PDAC seems context-dependent. When combined with standard chemotherapies (7, 8) or administered to obese *Kras*^*G12D*^ mice (bioRxiv 2025.05.20.655200), KD can improve survival and delay tumor progression. In contrast, KD accelerated tumor growth in nonobese *Kras*^*G12D*^ models (bioRxiv 2025.05.20.655200). These findings underscore the complexity of diet–tumor interactions and highlight that the preventive potential of KD in pancreatic cancer remains uncertain.

Recent transcriptomic analyses of healthy donor pancreata have revealed the unexpected presence of PanIN lesions, suggesting that early neoplastic lesions may be common and potentially amenable to dietary modulation (9). To explore how dietary lipid content influences early pancreatic tumorigenesis, we investigated the effects of KD, HFD, and low-fat diet (LFD) feeding on pancreatic disease progression using the *Ptf1a*^*CreERT2*^*;Kras*^*G12V*^ (*Acinar*^*KrasG12V*^) mouse model, which allows temporal control of oncogenic *Kras* activation in acinar cells. Mice were preconditioned with distinct dietary regimens prior to *Kras* activation and were monitored for survival, histopathologic progression, and inflammatory and molecular signaling.

Here, we demonstrate that dietary composition prior to oncogenic *Kras* activation profoundly influences pancreatic cancer trajectory. Although HFD was associated with accelerated disease progression, KD was correlated with unexpectedly aggressive pathology and reduced survival compared with LFD and standard diet (SD). Tumors from KD- and HFD-fed mice exhibited activation of the PI3K–Akt–mTOR and EGFR signaling pathways and were accompanied by a systemic proinflammatory, proangiogenic cytokine milieu. These findings suggest that dietary lipids can enhance oncogenic and inflammatory pathways in the pancreas, offering new insight into the dietary modulation of pancreatic cancer risk.

## Materials and Methods

### Animal experiments

All animal experiments were conducted in accordance with the guidelines approved by the Institutional Animal Care and Use Committee at the University of Texas Health Science Center at Houston and the University of Nebraska Medical Center. *Pft1a^Cre^*^*ERT2*^ mice (RRID:IMSR_JAX:019378) and wild-type (WT) C57BL/6J mice (RRID:IMSR_JAX:000664) were purchased from The Jackson Laboratory. Transgenic mice with *CAG-lox-GFP-stop-lox-Kras*^*G12V*^ (*cLGL-Kras*^*G12V*^) were received from Craig Logsdon, MD Anderson Cancer Center, Houston, TX (10). *Ptf1a^Cre^*^*ERT2*^ mice were crossed with *cLGL-Kras*^*G12V*^ mice to generate *Acinar*^*KrasG12V*^ mice, which express mutant *Kras* specifically in pancreatic acinar cells following Cre activation. *Acinar*^*KrasG12V*^ and WT control mice were littermates from the same breeding colony and were maintained under identical housing conditions throughout the study. Both male and female mice were included across all groups. Mice were maintained on a 12-hour light/dark cycle with *ad libitum* access to a SD (PicoLab Rodent Diet 20, LabDiet, #5053) until dietary intervention. The SD provides approximately 24.5% of energy from protein, 13.1% from fat, and 62.4% from carbohydrates.

### Tamoxifen dosing in *Acinar*^*KrasG12V*^ mice

To establish dosing conditions for oncogenic *Kras* activation, *Acinar*^*KrasG12V*^ mice (8–10 weeks old) received i.p. injections of tamoxifen (TAM) at doses of 1, 5, or 10 mg. Based on the dose administered, animals were designated as Kras^Low^ (1 mg), Kras^Mod^ (5 mg), or Kras^High^ (10 mg). Following treatment, mice were monitored for survival, and pancreatic tissues were collected for assessment of acinar Kras recombination, lesion development, and collagen deposition.

### Diet interventions

At 8 to 10 weeks of age, the mice were either maintained on the SD or randomly assigned to one of three experimental diets: KD (TD.160153, Teklad custom diet; 90% energy from fat), HFD (TD.160239, Teklad custom diet; 75% energy from fat, with a fat profile modified to better resemble the KD), or KD control diet (LFD; TD.150345, Teklad custom diet; 13% energy from fat). In total, the study included three WT and three *Acinar*^*KrasG12V*^ mice on SD, four WT and four *Acinar*^*KrasG12V*^ mice on the LFD, four WT and five *Acinar*^*KrasG12V*^ mice on the HFD, and five WT and five *Acinar*^*KrasG12V*^ mice on the KD. The calorie content and exact composition of each diet are provided in Supplementary Table S1. Mice were on the assigned diet for 1 month, after which both WT and *Acinar*^*KrasG12V*^ mice received a 1 mg intraperitoneal dose of TAM. TAM was administered following the establishment of diet-dependent metabolic states and at an identical dose across groups to ensure equivalent systemic metabolic effects. Body weight was recorded at the time of TAM induction and again 2 weeks after TAM for all groups. Mice were fed *ad libitum* to explore how diet may modulate the earliest stages of pancreatic neoplasia, and they remained on their respective diets until reaching the humane endpoint.

### Glucose tolerance test

The glucose tolerance test was carried out 10 days after TAM administration. Prior to the test, mice were subjected to a 6-hour fasting period. The glucose dose was calculated based on body weight after fasting (2.0 g of glucose per kilogram of body weight). Blood samples were obtained from the tail vein before glucose injection (baseline) and at 15, 30, 60, and 120 minutes afterward. Blood glucose concentrations were determined using a CONTOUR NEXT ONE blood glucose monitoring system.

### Hematoxylin and eosin staining

Formalin-fixed, paraffin-embedded tissues were sectioned and mounted on charged glass slides. Slides were deparaffinized in Histo-Clear, rehydrated through a graded ethanol to water, and stained with hematoxylin. Slides were then counterstained with eosin, dehydrated in ethanol, cleared in Histo-Clear, and mounted.

### IHC

Slides were baked at 60°C, deparaffinized in Histo-Clear, rehydrated through graded ethanol to PBS, and subjected to heat-mediated antigen retrieval using either citrate buffer (pH 6.0, Vector Laboratories, cat. #H-3300, RRID:AB_2336226) or Tris-EDTA buffer (pH 9.0, Abcam, cat. #AB93684). Sections were blocked for 1 hour at room temperature in 10% FBS prepared in PBST and then incubated overnight at 4°C with primary antibodies: cytokeratin 19 (CK19; Abcam, cat. #ab52625, RRID:AB_2281020), CD8α (Cell Signaling Technology, cat. #98941, RRID:AB_2756376), CD39 (Abcam, cat. #ab223842, RRID:AB_2889212), and CD73 (Cell Signaling Technology, cat. #13160, RRID:AB_2716625). The following day, slides were washed in PBS and incubated for 30 minutes at room temperature with biotinylated secondary antibodies (Vector Laboratories, cat. #BA-1000, RRID:AB_2313606). Signal detection was performed using the VECTASTAIN Elite ABC kit (Vector Laboratories, cat. #PK-4000, RRID:AB_2336818), followed by the DAB substrate (Vector Laboratories, cat. #SK-4100, RRID:AB_2336382). Sections were counterstained with hematoxylin, dehydrated through graded ethanol, cleared in Histo-Clear, and mounted.

### Trichrome staining

Connective tissue was visualized using the Abcam Trichrome Stain Kit (cat. #ab150686) according to the manufacturer’s protocol. Briefly, slides were deparaffinized in Histo-Clear, rehydrated through graded ethanol to distilled water, and incubated in preheated Bouin’s solution (60°C) for 60 minutes. After rinsing in tap water, sections were stained sequentially with Weigert’s iron hematoxylin (5 minutes), Biebrich Scarlet/acid fuchsin (15 minutes), phosphomolybdic–phosphotungstic acid (10–12 minutes), and aniline blue (10–20 minutes). Sections were then treated with acetic acid for 5 minutes, dehydrated through graded ethanol, cleared in Histo-Clear, and mounted.

### ImageJ analysis

Quantification of IHC and HE staining was performed using ImageJ software (RRID:SCR_003070). One section per mouse was analyzed, and 3 to 5 representative fields were selected depending on tissue size. For IHC, positive staining was identified using the color threshold tool, which normalized the signal across tissues. For analyses of acinar-to-ductal metaplasia (ADM), PanIN, or PDAC in hematoxylin and eosin (H&E)–stained sections, the freehand tool was similarly used to outline tissue regions of interest, and their relative areas were measured. H&E sections were also evaluated by a pathologist blinded to the treatment group to confirm histopathologic features.

### Reverse-phase protein assay

Reverse-phase protein assay (RPPA) analyses were performed on pancreatic lysates from WT mice (SD, *n* = 3; LFD, *n* = 2; KD, *n* = 3; HFD, *n* = 3) and *Acinar*^*KrasG12V*^ mice (SD, *n* = 3; LFD, *n* = 1; KD, *n* = 3; HFD, *n* = 3). Small pieces of whole frozen pancreatic tissue were homogenized in ice-cold lysis buffer [1% Triton X‐100, 50 mmol/L HEPES (pH 7.4), 150 mmol/L NaCl, 1.5 mmol/L MgCl_2_, 1 mmol/L EGTA, 100 mmol/L NaF, 10 mmol/L Na pyrophosphate, 1 mmol/L Na_3_VO_4_, 10% glycerol] supplemented with protease and phosphatase inhibitors. Lysates were centrifuged at 14,000 rpm for 10 minutes at 4°C, and the supernatant was collected. Protein concentration was measured by BCA assay and adjusted to 1.5 μg/μL with lysis buffer. Samples were combined with 4× SDS sample buffer [40% glycerol, 8% SDS, 0.25 mol/L Tris-HCl (pH 6.8)] containing β-mercaptoethanol at a 3:1 ratio (sample/buffer), boiled for 5 minutes, and stored at −80°C until analysis.

RPPA analysis was performed at the Functional Proteomics Core Facility at the University of Texas MD Anderson Cancer Center (RRID:SCR_016649). Protein lysates were serially diluted in five 2-fold steps and arrayed onto nitrocellulose-coated slides (Grace Bio-Labs) using a Quanterix 2470 Arrayer, generating 5,808 spots per slide. Each slide included experimental samples, standard lysates, and positive/negative controls. Each slide was probed with a validated primary antibody plus a biotin-conjugated secondary antibody. Signal was amplified with the Agilent GenPoint system and visualized by DAB colorimetric reaction. Slides were scanned using a Huron TissueScope, and spot intensities were quantified with Array-Pro Analyzer software (Media Cybernetics).

Relative protein expression was determined using RPPA SPACE software (MD Anderson Department of Bioinformatics and Computational Biology) through logistic “supercurve” fitting of the serial dilution data (11). A single fitted curve was generated for each antibody across all samples, with dilution step as the independent variable and signal intensity as the response. Protein levels were normalized for loading variation by bidirectional median centering (across samples for each antibody and across antibodies for each sample). Replicate-based normalization (RBN) using control samples was further applied to correct for interset variation, allowing direct comparison across different RPPA batches (12).

Differential protein expression across diet groups in WT and *Acinar*^*KrasG12V*^ mice was assessed using the limma package (RRID:SCR_010943) in R (version 4.5.2, RRID:SCR_001905). Multiple hypothesis testing correction was performed using the Benjamini–Hochberg method to control the false discovery rate. Volcano plots were generated using a significance threshold of *P* < 0.01 and |log_2_FC| = 0.2, where FC s fold change and visualized with ggplot2 (RRID:SCR_014601). KEGG pathway enrichment analysis was performed using proteins that were significantly upregulated (*P* < 0.05 and |log_2_FC| ≥ 0.10). Protein identifiers were mapped to mouse gene identifiers using org.Mm.eg.db (RRID:SCR_023488), and enrichment analysis was conducted using the clusterProfiler package (RRID:SCR_016884; enrichKEGG, organism = mmu) in R. Enriched pathways were ranked by enrichment *P* values, and the number of overlapping proteins contributing to each pathway is reported as the count (hits).

### Serum cytokine array

Serum cytokine and chemokine profiles were analyzed using the Proteome Profiler Mouse XL Cytokine Array kit (ARY028; R&D Systems), which detects the relative expression of 111 cytokines/chemokines simultaneously. The assay was performed according to the manufacturer’s protocol, and chemiluminescent signal intensity was used to determine relative expression levels. For this analysis, one biological replicate (serum sample) from each group: WT SD, *Acinar*^*KrasG12V*^ SD, WT KD, and *Acinar*^*KrasG12V*^ KD, was included. Imaging was performed on a Bio-Rad ChemiDoc instrument (RRID:SCR_019037). A heatmap of serum cytokine expression was generated using the pheatmap (RRID:SCR_016418) package in R.

### Serum acetate, β-hydroxybutyrate, amylase, and aspartate aminotransferase levels

Serum acetate levels were quantified using the Acetate Colorimetric Assay Kit (MilliporeSigma, cat. #MAK086). Serum β-hydroxybutyrate (β-OHB) levels were measured using the β-Hydroxybutyrate Assay Kit (MilliporeSigma, cat. #MAK041). Serum amylase levels were quantified using the Pointe Liquid Amylase (CNPG3) Reagent Set (MedTest DX, cat. #23-666-111). Aspartate aminotransferase (AST) levels were measured using the AST (SGOT) – IFCC Liquid Reagent Set (Teco Diagnostics, cat. #A559-150). All assays were performed according to the manufacturers’ instructions.

### Statistical analysis

Statistical comparisons were performed in GraphPad Prism (RRID:SCR_002798) using unpaired *t* tests and one- or two-way ANOVA, followed by Sidak or Tukey multiple comparisons tests. For survival analyses, Kaplan–Meier methods were used to estimate survival distributions, and comparisons between groups were made using the log-rank (Mantel–Cox) test.

## Results

### TAM dose determines the extent of acinar *Kras* recombination and influences pancreatic disease progression

**Figure 1.**
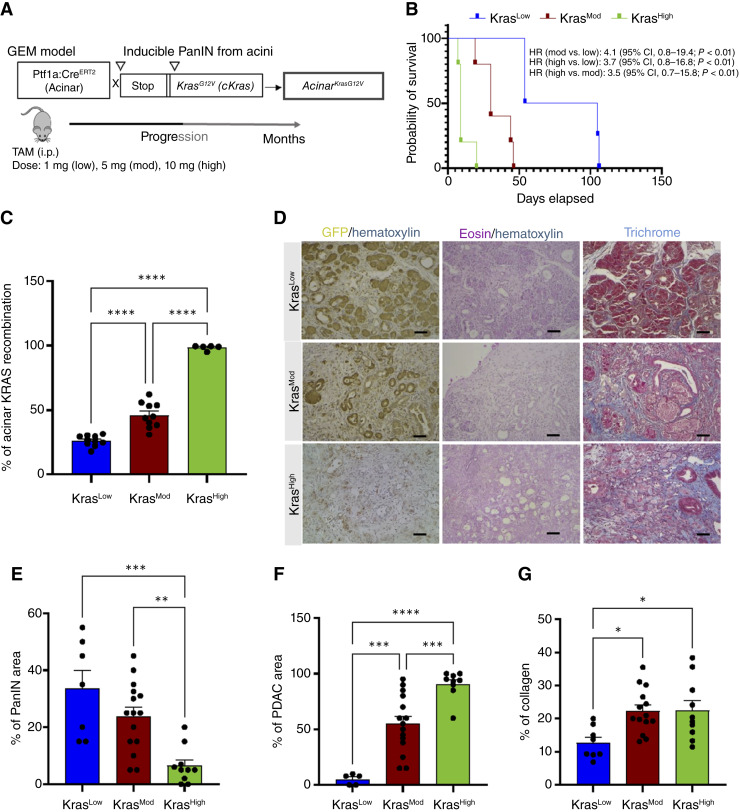
Dose-dependent effects of TAM on *Kras* recombination, survival, and pancreatic pathology in *Acinar*^*KrasG12V*^ mice. **A,** Schematic of the experimental plan showing the effect of varying TAM doses (1 mg, Kras^Low^; 5 mg, Kras^Mod^; 10 mg, Kras^High^) on *Kras* recombination efficiency in *Acinar*^*KrasG12V*^ mice. **B,** Kaplan–Meier survival curve showing the survival of *Acinar*^*KrasG12V*^ mice treated with different TAM doses. **C,** Percentage of acinar *Kras* recombination in Kras^Low^, Kras^Mod^, and Kras^High^ mice, as assessed by GFP loss. **D,** Representative images showing H&E and trichrome staining of pancreatic tissue from Kras^Low^, Kras^Mod^, and Kras^High^ mice. Scale bars are 50 μmol/L. Percentage of (**E**) PanIN lesions, (**F**) PDAC, and (**G**) collagen deposition in Kras^Low^, Kras^Mod^, and Kras^High^ mice. Statistical significance was determined by one-way ANOVA with the Tukey multiple comparison test. *, *P* < 0.05; **, *P* < 0.01; ***, *P* < 0.001; ****, *P* < 0.0001.

Previously, our lab showed that TAM dose-dependent expression of *Kras*^*G12V*^ in pancreatic ducts leads to both early- and late-stage PanINs and invasive PDAC (13). Here, we sought to test whether TAM dosage correlates with the degree of oncogenic *Kras* activation in acinar cells and subsequent disease progression. *Acinar*^*KrasG12V*^ mice were treated with low (1 mg, Kras^Low^), moderate (5 mg, Kras^Mod^), or high (10 mg, Kras^High^) doses of TAM. As shown in Fig. 1A, TAM treatment activates *Ptf1a*^*Cre*^^*ERT2*^ in the acinar cells, inducing recombination at the LSL-*Kras*^*G12V*^ allele. Kaplan–Meier survival analysis revealed a dose-dependent effect of TAM on survival. Kras^Low^ mice exhibited prolonged survival, with some surviving beyond 100 days, whereas Kras^Mod^ and Kras^High^ mice showed markedly reduced survival, with complete mortality by ∼46 and ∼20 days, respectively. Hazard ratio analysis confirmed a significantly increased risk of death in Kras^Mod^ and Kras^High^ mice compared with Kras^Low^ mice (*P* < 0.01; Fig. 1B). Recombination efficiency within acinar cells, assessed by loss of green fluorescent protein (GFP) expression, also showed a clear dose-dependent effect (Fig. 1C and D). Kras^Low^ mice exhibited ∼26% recombination, Kras^Mod^ mice ∼46%, and Kras^High^ mice nearly complete recombination (∼98%) with all comparisons highly significant (*P* < 0.0001). Histopathologic assessment further demonstrated that both the type and severity of pancreatic lesions were associated with TAM dose (Fig. 1D–G). Kras^Low^ mice showed increased PanIN lesions compared with Kras^High^ mice (*P* < 0.001; Fig. 1E). Conversely, invasive PDAC lesions were more prominent in Kras^High^ mice, significantly higher than in Kras^Mod^ (*P* < 0.001) and Kras^Low^ (*P* < 0.0001) mice (Fig. 1F). Trichrome staining showed elevated collagen deposition in Kras^Mod^ (*P* < 0.05) and Kras^High^ (*P* < 0.05) mice compared with Kras^Low^ mice (Fig. 1G). Together, these results demonstrate that TAM dose determines the extent of acinar *Kras*^*G12V*^ recombination and is closely associated with the trajectory of disease progression from PanIN to PDAC. Consequently, the 1 mg dose was selected for subsequent dietary intervention studies because it induces partial recombination, predominantly PanIN lesions, and extended survival, providing conditions well suited for evaluating how different dietary exposures influence the initiation and progression of early pancreatic neoplasia.

### KD is associated with reduced survival in *Acinar*^*KrasG12V*^ mice and worsened glucose intolerance in WT controls

**Figure 2.**
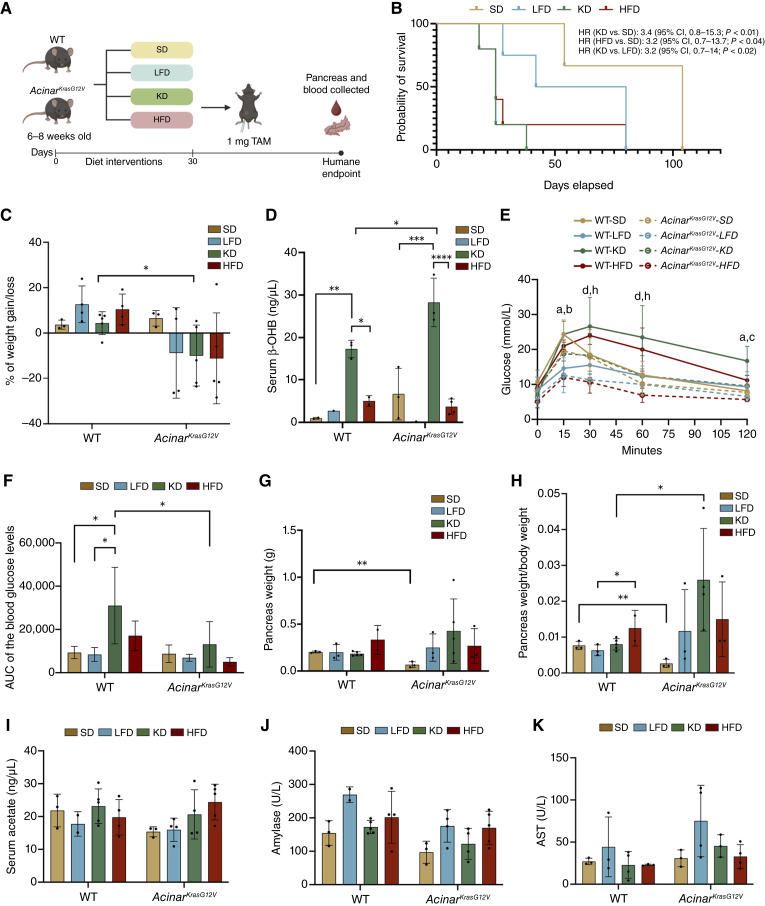
KD is associated with reduced survival in *Acinar*^*KrasG12V*^ mice and increased glucose intolerance in WT controls. **A,** Experimental design. WT and *Acinar*^*KrasG12V*^ mice (8–10 weeks old) were fed SD, LFD, KD, or HFD for 30 days and then received 1 mg of TAM. Pancreas and serum were collected at the humane endpoint. **B,** Kaplan–Meier survival curves showing the probability of survival for *Acinar*^*KrasG12V*^ mice under different dietary interventions. **C,** Percentage change in body weight at 2 weeks after TAM relative to the day of TAM induction in WT and *Acinar*^*KrasG12V*^ mice. **D,** Serum β-OHB levels at the endpoint in WT and *Acinar*^*KrasG12V*^ mice under each diet. **E,** Blood glucose levels and (**F**) AUC measured 3 weeks after TAM induction in the WT and *Acinar*^*KrasG12V*^ mice. **G,** Absolute pancreas weight and (**H**) pancreas weight relative to body weight at the endpoint across dietary groups in WT and *Acinar*^*KrasG12V*^ mice. Serum levels of (**I**) acetate, (**J**) amylase, and (**K**) AST at the endpoint in WT and *Acinar*^*KrasG12V*^ mice across all dietary groups. Statistical analyses were performed using unpaired *t* tests (**C**), one-way ANOVA with Sidak test (**D**), one-way ANOVA with group significance denoted by letter pairs (a = WT-SD, b = WT-LFD, c = WT-KD, d = WT-HFD, e = *Acinar*^*KrasG12V*^-SD, f = *Acinar*^*KrasG12V*^-LFD, g = *Acinar*^*KrasG12V*^-KD, h = *Acinar*^*KrasG12V*^-HFD; **E**), one-way ANOVA with Tukey test and two-way ANOVA with Sidak test (**F**), and unpaired *t* test and one-way ANOVA with Tukey test (**G** and **H**). Data are presented as mean ± SEM. *, *P* < 0.05; **, *P* < 0.01; ***, *P* < 0.001; ****, *P* < 0.0001.

To determine whether dietary composition influences PDAC development, WT and *Acinar*^*KrasG12V*^ mice, aged 8 to 10 weeks, were either maintained on SD or randomly assigned to one of three diets: LFD, HFD, or KD. After 1 month on the assigned diets, *Acinar*^*KrasG12V*^ mice were treated with 1 mg TAM and were monitored for survival and body weight (Fig. 2A). KD-fed *Acinar*^*KrasG12V*^ mice had the shortest median survival (26 ± 7 days), significantly lower than the SD-fed (87 ± 29 days, *P* = 0.02) and LFD-fed mice (57 ± 27 days, *P* = 0.02). Similarly, HFD feeding was associated with reduced survival (35 ± 25 days, *P* = 0.05) compared with SD (Fig. 2B). These findings indicate that both KD and HFD are associated with accelerated disease progression and earlier mortality. At the humane endpoint, KD-fed *Acinar*^*KrasG12V*^ mice exhibited significant weight loss compared with KD-fed WT mice (*P* = 0.05; Fig. 2C). As expected, KD feeding significantly elevated serum β-OHB in WT mice compared with SD (*P* = 0.003) and HFD (*P* = 0.02), with a similar trend in *Acinar*^*KrasG12V*^ mice (KD vs. SD and HFD, *P* = 0.0001). Furthermore, KD-fed *Acinar*^*KrasG12V*^ mice exhibited higher β-OHB than KD-fed WT controls (*P* = 0.03; Fig. 2D). Blood glucose levels measured 3 weeks after the TAM induction in the WT and *Acinar*^*KrasG12V*^ mice are shown in Fig. 2E. In WT mice, KD caused impaired glucose tolerance, reflected by a significantly increased area under the curve (AUC) compared with SD (*P* = 0.03) and LFD (*P* = 0.01). When comparing across genotypes, *Acinar*^*KrasG12V*^ mice displayed lower glucose AUC than WT mice under KD (*P* = 0.02; Fig. 2F). KD-fed *Acinar*^*KrasG12V*^ mice showed increased pancreas weight and a significantly higher pancreas–to–body weight ratio relative to WT controls, whereas SD-fed *Acinar*^*KrasG12V*^ mice exhibited significantly reduced pancreas weight and a lower pancreas–to–body weight ratio compared with WT mice (Fig. 2G and H). No significant differences were observed in serum acetate, amylase, or AST levels across diets in either genotype (Fig. 2I–K).

### KD and HFD are associated with increased fibrosis and progression to PDAC in nonobese *Acinar*^*KrasG12V*^ mice

**Figure 3.**
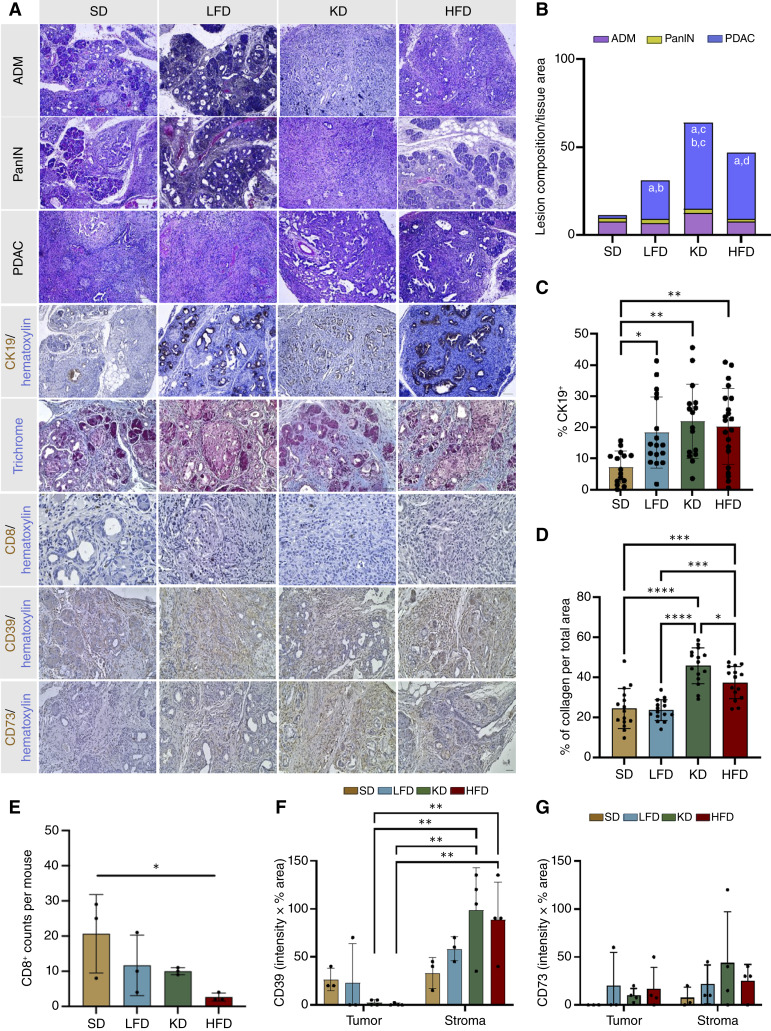
KD and HFD are associated with increased fibrosis and progression to PDAC in *Acinar*^*KrasG12V*^ mice. **A,** Representative images of pancreatic tissue from SD, LFD, KD, and HFD-fed *Acinar*^*KrasG12V*^ mice showing ADM, PanIN lesions, PDAC, CK19 immunostaining, trichrome staining, and CD8, CD39, and CD73 expression. Scale bars, 50 μm. **B,** Stacked bar graph depicting the percentage of ADM, PanIN, and PDAC across dietary groups. Quantification of (**C**) CK19^+^ epithelial area, (**D**) collagen deposition (trichrome^+^ area), and (**E**) CD8^+^ T-cell counts per mouse in SD, LFD, KD, and HFD-fed *Acinar*^*KrasG12V*^ mice. **F,** CD39 expression (intensity × % area) and (**G**) CD73 expression (intensity × % area) quantified separately in tumor and stromal compartments across diets. Statistical analyses were performed using one-way ANOVA with group significance denoted by letter pairs (a = SD, b = LFD, c = KD, and d = HFD; **B**), one-way ANOVA with Sidak test (**C–E**), and two-way ANOVA with Sidak test (**F** and **G**). Data are presented as mean ± SEM. *, *P* < 0.05; **, *P* < 0.01; ***, *P* < 0.001; ****, *P* < 0.0001.

As KD- and HFD-fed *Acinar*^*KrasG12V*^ mice exhibited the worst survival outcomes, we next examined tissue-level changes using histologic and IHC analyses. Figure 3A shows the representative images of ADM, PanIN, and PDAC, CK19, trichrome, CD8, CD39, and CD73 staining in pancreatic tissue sections from *Acinar*^*KrasG12V*^ mice under different diets. Pathologic evaluation revealed distinct differences in lesion grade and tissue architecture (Table 1). The proportion of pancreas replaced by invasive, sarcomatoid-like PDAC was markedly higher in KD-fed *Acinar*^*KrasG12V*^ mice compared with SD-fed mice, which had moderately differentiated neoplasia (*P* < 0.0001), and LFD-fed mice that presented with well to moderately differentiated PDAC (*P* = 0.01). HFD-fed *Acinar*^*KrasG12V*^ mice also showed an increase in poorly differentiated PDAC compared with SD (*P* = 0.0007; Fig. 3B). These data indicate that both KD and HFD are associated with the development of aggressive and invasive PDAC in *Acinar*^*KrasG12V*^ mice. The percentage of CK19^+^ area, indicating that ductal-like differentiation was elevated in LFD-, KD-, and HFD-fed *Acinar*^*KrasG12V*^ mice compared with SD (*P* = 0.02, *P* = 0.002, and *P* = 0.003, respectively; Fig. 3C). Fibrosis, assessed by collagen^+^ area, was markedly increased in KD-fed *Acinar*^*KrasG12V*^ mice, which exhibited the strongest fibrotic response (*P* = 0.005 vs. SD; *P* = 0.0002 vs. LFD). Compared with LFD, HFD-fed *Acinar*^*KrasG12V*^ mice also showed significantly higher fibrosis (*P* = 0.01; Fig. 3D). Furthermore, there were significantly fewer CD8^+^ T cells in HFD-fed *Acinar*^*KrasG12V*^ mice compared with SD, suggesting that HFD may be associated with reduced antitumor immune infiltration within the tumor microenvironment (Fig. 3E). Stromal CD39 expression was significantly higher in KD- and HFD-fed *Acinar*^*KrasG12V*^ mice compared with their respective tumor compartments, whereas CD73 expression showed no significant differences across diets (Fig. 3F and G). Together, these findings demonstrate that dietary composition is associated with pancreatic lesion severity, fibrosis, and the immune cell landscape in *Acinar*^*KrasG12V*^ mice, with KD and HFD corresponding to more aggressive disease phenotypes.

**Table 1.** Pathologic evaluation of pancreatic tissue sections from *Acinar*^*KrasG12V*^ mice under different diets.

Diet	Pathologic description
SD	Predominantly normal acinar architecture with residual acini visible; minimal evidence of malignancy; mild to moderate inflammatory infiltrate; moderately differentiated lesions; islet cell hyperplasia
LFD	Areas of malignancy involving acinar regions; mild inflammation; overall well to moderately differentiated lesions
KD	Presence of sarcomatoid transformation, mild to moderate inflammation, neutrophil infiltration leading to cell death and release of DAMPs; increased stemness observed
HFD	Marked malignancy with poorly differentiated lesions; minimal inflammation observed

### HFDs and KDs are associated with reprogramming of tumor signaling and systemic cytokine profiles

**Figure 4.**
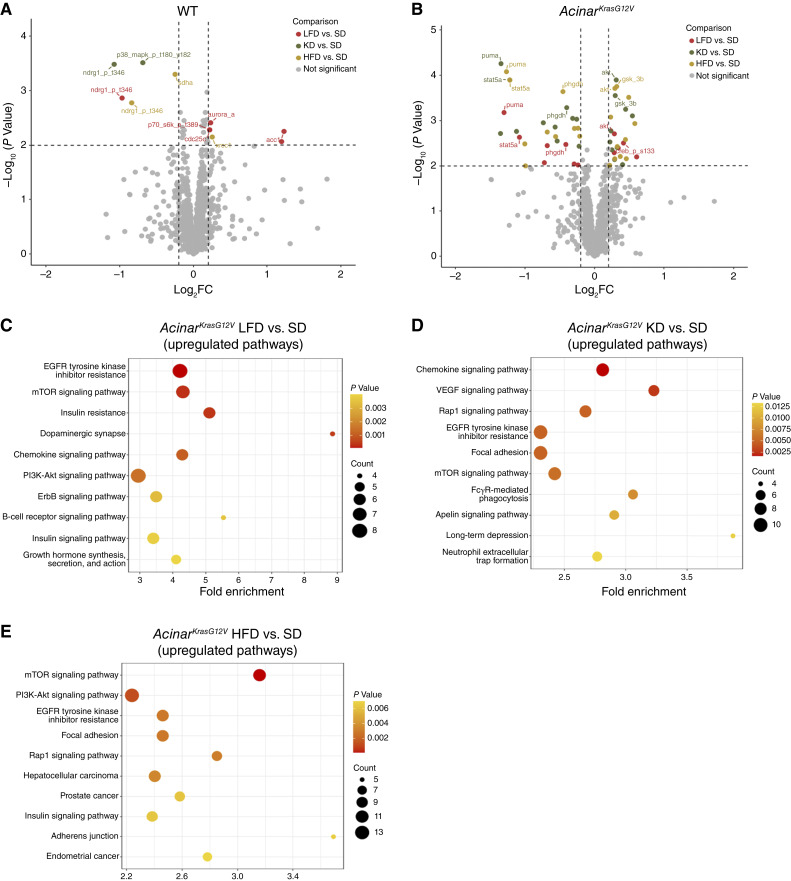
Diet-induced alterations in pancreatic signaling pathways in WT and *Acinar*^*KrasG12V*^ mice. Volcano plot illustrating differential protein expression in (**A**) WT mice and (**B**) *Acinar*^*KrasG12V*^ mice fed LFD, KD, or HFD relative to SD. Pancreatic lysates were analyzed by RPPA (WT: SD, *n* = 3; LFD, *n* = 2; KD, *n* = 3; HFD, *n* = 3 and *Acinar*^*KrasG12V*^: SD, *n* = 3; LFD, *n* = 1; KD, *n* = 3; HFD, *n* = 3) using the limma package. Significant proteins are highlighted (LFD vs. SD: red, KD vs. SD: green, and HFD vs. SD: yellow), and nonsignificant proteins are shown in gray. The *x*-axis represents log_2_ FC, and the *y*-axis shows −log_10_(*P*) values. Dotted lines indicate *P* = 0.01 and |log_2_FC| = 0.2 thresholds. KEGG pathway enrichment analysis of upregulated proteins (*P* < 0.05 and |log_2_FC| ≥ 0.10) in *Acinar*^*KrasG12V*^ mice for each comparison: (**C**) LFD vs. SD, (**D**) KD vs. SD, and (**E**) HFD vs. SD. Bubble plots display the top enriched pathways ranked by fold enrichment (*x*-axis). Bubble size (counts) corresponds to the number of upregulated proteins contributing to each pathway, and bubble color reflects pathway significance (*P* value).

Given the more aggressive histopathology observed in KD- and HFD-fed mice, we next sought to determine the molecular signaling pathways underlying these diet-induced effects. We performed RPPA analysis of whole pancreatic tissues to assess local alterations in oncogenic and inflammatory signaling (Supplementary Table S2). Volcano plots highlight significant protein alterations (*P* < 0.01, |log_2_FC| > 0.2) in LFD-, KD-, and HFD-fed mice compared with SD-fed controls in WT mice (Fig. 4A) and in the *Acinar*^*KrasG12V*^ model (Fig. 4B). Compared with SD-fed *Acinar*^*KrasG12V*^ mice, LFD-fed mice exhibited 12 altered proteins (7 downregulated and 5 upregulated), KD-fed mice showed 18 altered proteins (8 downregulated and 10 upregulated), and HFD-fed mice displayed 21 altered proteins (10 downregulated and 11 upregulated). Several proteins were commonly dysregulated in both KD- and HFD-fed groups, including upregulation of Akt, GSK3β, phosphorylated S6 (Ser235/236), phosphorylated AMPKα (Thr172), paxillin, and YTHDF2 and downregulation of PUMA, STAT5A, c-kit, PHGDH, FASN, and ASNS. Moreover, LFD-fed mice showed upregulation of Akt and phosphorylated CREB (Ser133) and downregulation of PUMA, STAT5A, and c-Kit (Supplementary Table S3; Fig. 4B). To identify biological processes affected by these proteomic changes, KEGG pathway enrichment analysis was performed. The top 10 enriched KEGG pathways for LFD-, HFD-, and KD-fed *Acinar*^*KrasG12V*^ mice compared with SD are shown in Fig. 4C–E, respectively. Across all diet groups, several pathways were commonly upregulated, including EGFR tyrosine kinase inhibitor resistance, chemokine signaling, mTOR signaling, PI3K–Akt signaling, insulin resistance, Rap1 signaling, and VEGF signaling pathways (Supplementary Table S4; Fig. 4C–E).

**Figure 5.**
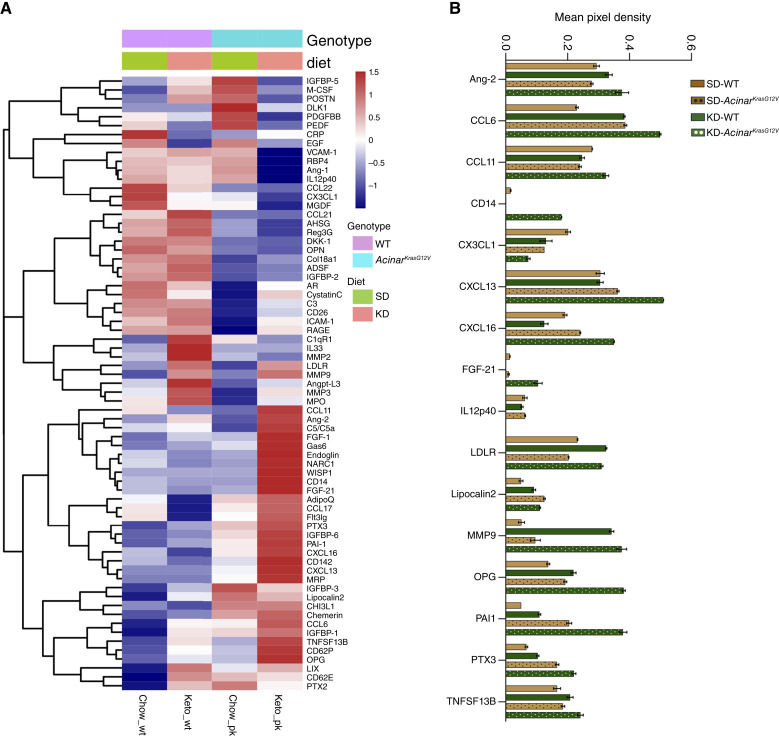
KD is associated with systemic inflammatory and angiogenic cytokine responses in WT and *Acinar*^*KrasG12V*^ mice. **A,** Heatmap depicting serum cytokine and chemokine expression across WT and *Acinar*^*KrasG12V*^ mice fed SD or KD. Cytokines/chemokines are hierarchically clustered by expression pattern. Red indicates higher abundance, whereas blue indicates lower abundance. **B,** Bar graph showing the mean pixel density of cytokines/chemokines in serum from SD-fed WT, KD-fed WT, SD-fed *Acinar*^*KrasG12V*^, and KD-fed *Acinar*^*KrasG12V*^ mice. One biological replicate was analyzed per group.

To determine systemic factors underlying accelerated disease progression in KD-fed *Acinar*^*KrasG12V*^ mice, we profiled serum cytokines in SD- and KD-fed WT and *Acinar*^*KrasG12V*^ mice (Supplementary Table S5). The heatmap (Fig. 5A) revealed a shift in serum cytokine levels in KD-fed groups, with the most profound changes observed in *Acinar*^*KrasG12V*^ mice. The serum levels of Ang-2, CCL6, LDLR, MMP-9, PAI-1, PTX3, TNFSF13B, and OPG were increased in KD-fed mice and were further elevated in KD-fed *Acinar*^*KrasG12V*^ mice compared with their respective SD-fed controls (Fig. 5B). Additionally, CCL11, CD14, and FGF-21 were elevated, whereas CX3CL1 and IL12p40 were reduced in KD-fed *Acinar*^*KrasG12V*^ mice relative to SD-fed counterparts (Fig. 5B). Collectively, these findings indicate that the KD is associated with a systemic proinflammatory and proangiogenic cytokine milieu, coinciding with the activation of PI3K–Akt–mTOR and EGFR signaling in the pancreas. Together, these tissue and serum-level alterations provide mechanistic insight into how a lipid-rich diet may contribute to pancreatic tumor progression in *Acinar*^*KrasG12V*^ mice.

## Discussion

Dietary composition is increasingly recognized as a modifiable factor that can influence pancreatic cancer risk, yet its impact on the earliest stages of KRAS-driven transformation has remained unclear. Using an inducible acinar-specific *Kras* model, we found that pretumor dietary exposure was associated with substantial differences in the pancreatic disease trajectory. Both the KD and HFD were associated with accelerated disease progression, increased invasive PDAC, fibrosis, and reduced survival compared with SD and LFD. Previous studies have shown that acinar cells possess plasticity in response to inflammation and *Kras*^*G12D*^ mutations (14–17), and our findings expand understanding of how acinar cells respond to dietary changes in the context of *Kras*^*G12V*^ mutations. These findings indicate that macronutrient balance prior to oncogenic activation is associated with how pancreatic tissue responds to KRAS signaling, with lipid-rich diets corresponding to conditions that favor aggressive neoplasia.

More than 90% of human PDACs harbor activating *KRAS* mutations, establishing KRAS as a central driver of disease initiation (18, 19). Although PDAC exhibits ductal histology, experimental and human studies support acinar cells as a major cell of origin through KRAS-dependent ADM (20, 21). Compared with commonly used PDAC models, including KPC models of advanced and metastatic disease and KC models with constitutive *Kras*^*G12D*^ activation in broader pancreatic lineages during development, our TAM-inducible acinar-specific *Kras*^*G12V*^ model provides temporal control of oncogenic activation (22, 23). This feature makes the model particularly well-suited for investigating how environmental factors such as diet may modulate KRAS signaling during early pancreatic tumorigenesis.

Diets used in this study represent distinct metabolic states that may influence pancreatic cancer risk and progression. KD, characterized by very low carbohydrate and high fat content, induces nutritional ketosis and is increasingly investigated as a metabolic intervention in cancer although its effects in PDAC remain context-dependent (bioRxiv 2025.05.20.655200; refs. 5, 7, 8). In contrast, HFD reflects obesogenic Western dietary patterns associated with obesity, metabolic dysfunction, and increased pancreatic cancer risk (24–26). LFD enabled evaluation of minimal fat intake with higher carbohydrate exposure, allowing comparison across divergent macronutrient states. SD served as the physiologic reference diet, providing a metabolic baseline for comparison with experimental diets. Similar macronutrient formulations have been widely used in prior PDAC studies, facilitating cross-study comparisons (bioRxiv 2025.05.20.655200; refs. 2–4, 7, 8).

The detrimental impact of HFD is consistent with prior work showing that obesogenic high-fat feeding promotes acinar injury, ADM, PanIN formation, and PDAC progression in *Kras*^*G12D*^ and *Kras*^*G12V*^*/Trp53*-loss mouse models (bioRxiv 2026.02.10.703309; refs. 2, 3). Furthermore, our results align with emerging evidence that KD can accelerate PDAC development in nonobese *Kras*-mutant models while delaying tumor progression in obesity-associated settings (bioRxiv 2025.05.20.655200). This context dependency suggests that metabolic state rather than KD itself determines whether very low–carbohydrate, high-fat intake is protective or harmful. Importantly, because KD and LFD represent metabolically extreme dietary states, the observed effects may reflect systemic metabolic stress rather than the effects of fat or carbohydrate content alone. In our study, KD-fed WT developed significant glucose intolerance, a phenotype also reported in nonobese rodents fed long-term KD (27). These findings are consistent with impaired insulin sensitivity although insulin and C-peptide were not measured. Given that insulin resistance can amplify PI3K–Akt–mTOR signaling and that this pathway cooperates with KRAS to drive early neoplastic progression, diet-induced metabolic stress may have contributed to the enhanced disease in KD-fed mice (28, 29). Additionally, the lower glucose AUC observed in KD *Acinar*^*KrasG12V*^ mice relative to WT controls may reflect tumor-associated alterations in systemic glucose utilization rather than improved metabolic function.

Although LFD was less detrimental than KD or HFD, our findings parallel previous work demonstrating that carbohydrate-rich diets can also promote early pancreatic lesions (4). The LFD used in our study (77% carbohydrate) is compositionally similar to the HCD evaluated by Zhu and colleagues (4), who reported shortened survival and increased ADM, inflammation, and PanIN development in *Kras*^*G12D*^ mice. Their study suggested that sucrose-rich carbohydrate sources may be particularly associated with harmful effects, consistent with epidemiologic observations linking high intake of simple sugars or fructose-sweetened beverages to pancreatic cancer risk (30). Together, our results support the concept that both excessive dietary fat and high proportions of rapidly metabolized carbohydrates are associated with worsened early pancreatic neoplasia although lipid-rich diets exerted the strongest effects.

KD and HFD were associated with increased collagen deposition and elevated stromal CD39 expression. CD39, together with CD73, controls extracellular adenosine production and is associated with immunoregulatory signaling (31). Clinical and preclinical studies show that stromal CD39 and tumoral CD73 correlate with poor prognosis in PDAC and can negate the otherwise favorable impact of CD8^+^ tumor infiltration (32). Thus, the stromal and immune alterations observed in KD- and HFD-fed mice are consistent with a microenvironment that may support more advanced disease. Furthermore, recent work demonstrates that β-HB can enhance tumor invasiveness by stabilizing the epithelial–mesenchymal transition regulator Snail through lysine β-hydroxybutyrylation (33). This epigenetic mechanism offers a plausible link between increased ketone body exposure and the enhanced ductal transformation and fibrosis observed in KD-fed *Acinar*^*KrasG12V*^ mice. At the systemic level, KD triggered a proinflammatory and proangiogenic cytokine profile, with elevated Ang-2, MMP-9, PAI-1, PTX3, and TNFSF13B, among others. These cytokines are known to support extracellular matrix remodeling, tissue invasion, and angiogenesis, suggesting that KD may contribute to a tumor-promoting systemic environment (21, 34, 35).

Proteomic profiling provided additional insight into convergent diet-driven mechanisms. KD and HFD were associated with the activation of multiple oncogenic pathways, including PI3K–Akt–mTOR, EGFR, chemokine signaling, and insulin resistance pathways that are closely tied to PDAC initiation and progression. These findings contrast with reports in established tumor models in which KD suppresses AKT and ERK signaling, particularly when combined with chemotherapy (7). This discrepancy underscores the importance of disease stage: Early PanIN lesions may exploit high lipid availability and ketone bodies to fuel growth, whereas established tumors may respond differently to the same nutritional conditions and when coupled with chemotherapy. Thus, the timing of dietary exposure relative to oncogenic events is likely critical in determining biological outcomes. RPPA and cytokine analyses were performed using a limited number of biological replicates and are therefore considered exploratory and hypothesis-generating. Accordingly, pathway-level findings from these datasets should be regarded as preliminary. Additionally, as no vehicle-treated controls were included in the study, it remains difficult to distinguish diet-specific effects from potential interactions between TAM and diet-induced metabolic states.

Future functional studies should evaluate whether inhibition of PI3K–Akt–mTOR signaling, modulation of CD39-associated immune pathways, or blockade of key cytokine networks can alter the accelerated PanIN-to-PDAC progression observed in KD- and HFD-fed mice. Such studies will help determine whether these changes directly drive diet-associated pancreatic neoplasia or are simply associated with disease progression.

In summary, our findings demonstrate that dietary lipid content before oncogenic *Kras* activation is strongly associated with differences in the initiation and progression of pancreatic cancer. Both KD and HFD were associated with fibrosis, immune remodeling, increased CD39 stromal expression, systemic inflammation, and activation of key oncogenic pathways, collectively corresponding to accelerated progression from PanIN to PDAC. These results contribute to a growing understanding of how diet shapes early neoplastic evolution and underscore the need for careful consideration of high-fat or ketogenic dietary patterns among individuals at elevated risk for pancreatic cancer.

## Supplementary Material

Supplementary Table S2 Supplementary Table S2 shows the RPPA raw data files.

Supplementary Table S3 Supplementary Table S3 shows the RPPA limma files.

Supplementary Table S4 Supplementary Table S4 shows the comprehensive KEGG pathway analysis.

Supplementary Table S5 Supplementary Table S5 shows the serum profiling raw data file.

Supplementary Table S1 Supplementary Table S1 shows the diet compositions.

## Data Availability

RPPA and serum proteome profiler raw data are available in Supplementary Tables S2 and S5, respectively. All other data generated in this study are available from the corresponding author upon request.
